# Community-based antiretroviral therapy versus standard clinic-based services for HIV in South Africa and Uganda (DO ART): a randomised trial

**DOI:** 10.1016/S2214-109X(20)30313-2

**Published:** 2020-09-21

**Authors:** Ruanne V Barnabas, Adam A Szpiro, Heidi van Rooyen, Stephen Asiimwe, Deenan Pillay, Norma C Ware, Torin T Schaafsma, Meighan L Krows, Alastair van Heerden, Philip Joseph, Maryam Shahmanesh, Monique A Wyatt, Kombi Sausi, Bosco Turyamureeba, Nsika Sithole, Susan Morrison, Adrienne E Shapiro, D Allen Roberts, Katherine K Thomas, Olivier Koole, Anna Bershteyn, Peter Ehrenkranz, Jared M Baeten, Connie Celum

**Affiliations:** a Department of Global Health, University of Washington, Seattle, WA, USA; b Division of Allergy and Infectious Diseases, University of Washington, Seattle, WA, USA; c Department of Epidemiology, University of Washington, Seattle, WA, USA; d Department of Biostatistics, University of Washington, Seattle, WA, USA; e Vaccine and Infectious Diseases Division, Fred Hutchinson Cancer Research Center, Seattle, WA, USA; f Human Sciences Research Council, Sweetwaters, KwaZulu-Natal, South Africa; g MRC/Wits Developmental Pathways for Health Research Unit, University of the Witwatersrand, Johannesburg, South Africa; h Integrated Community-Based Initiatives, Kabwohe, Uganda; i Africa Health Research Institute, KwaZulu-Natal, South Africa; j Harvard Medical School, Boston, MA, USA; k London School of Hygiene & Tropical Medicine, London, UK; l New York University School of Medicine, New York, NY, USA; m The Bill & Melinda Gates Foundation, Seattle, WA, USA

## Abstract

**Background:**

Community-based delivery of antiretroviral therapy (ART) for HIV, including ART initiation, clinical and laboratory monitoring, and refills, could reduce barriers to treatment and improve viral suppression, reducing the gap in access to care for individuals who have detectable HIV viral load, including men who are less likely than women to be virally suppressed. We aimed to test the effect of community-based ART delivery on viral suppression among people living with HIV not on ART.

**Methods:**

We did a household-randomised, unblinded trial (DO ART) of delivery of ART in the community compared with the clinic in rural and peri-urban settings in KwaZulu-Natal, South Africa and the Sheema District, Uganda. After community-based HIV testing, people living with HIV were randomly assigned (1:1:1) with mobile phone software to community-based ART initiation with quarterly monitoring and ART refills through mobile vans; ART initiation at the clinic followed by mobile van monitoring and refills (hybrid approach); or standard clinic ART initiation and refills. The primary outcome was HIV viral suppression at 12 months. If the difference in viral suppression was not superior between study groups, an a-priori test for non-inferiority was done to test for a relative risk (RR) of more than 0·95. The cost per person virally suppressed was a co-primary outcome of the study. This study is registered with ClinicalTrials.gov, NCT02929992.

**Findings:**

Between May 26, 2016, and March 28, 2019, of 2479 assessed for eligibility, 1315 people living with HIV and not on ART with detectable viral load at baseline were randomly assigned; 666 (51%) were men. Retention at the month 12 visit was 95% (n=1253). At 12 months, community-based ART increased viral suppression compared with the clinic group (306 [74%] *vs* 269 [63%], RR 1·18, 95% CI 1·07–1·29; p_superiority_=0·0005) and the hybrid approach was non-inferior (282 [68%] *vs* 269 [63%], RR 1·08, 0·98–1·19; p_non-inferiority_=0·0049). Community-based ART increased viral suppression among men (73%, RR 1·34, 95% CI 1·16–1·55; p_superiority_<0·0001) as did the hybrid approach (66%, RR 1·19, 1·02–1·40; p_superiority_=0·026), compared with clinic-based ART (54%). Viral suppression was similar for men (n=156 [73%]) and women (n=150 [75%]) in the community-based ART group. With efficient scale-up, community-based ART could cost US$275–452 per person reaching viral suppression. Community-based ART was considered safe, with few adverse events.

**Interpretation:**

In high and medium HIV prevalence settings in South Africa and Uganda, community-based delivery of ART significantly increased viral suppression compared with clinic-based ART, particularly among men, eliminating disparities in viral suppression by gender. Community-based ART should be implemented and evaluated in different contexts for people with detectable viral load.

**Funding:**

The Bill & Melinda Gates Foundation; the University of Washington and Fred Hutch Center for AIDS Research; the Wellcome Trust; the University of Washington Royalty Research Fund; and the University of Washington King K Holmes Endowed Professorship in STDs and AIDS.

## Introduction

Of the 37 million people estimated to be living with HIV globally, approximately 62% are on life-saving antiretroviral therapy (ART) and 53% are virally suppressed.^1^ Detectable viral load increases HIV-associated morbidity and mortality^2^, ^3^ and increases HIV transmission.^4^ Standard clinic-based delivery of ART, including a growing number of streamlined delivery models for stable patients^5^ who have suppressed viral load after 12 months of treatment, have successfully expanded ART coverage globally. However, people with detectable viral load are usually not eligible for streamlined services, such as fast-track ART refills. In the generalised epidemic setting in southern and eastern Africa, where the majority of people living with HIV reside, the overall rate of viral suppression is 54% in South Africa and 64% in Uganda.^1^ Further, men are less likely to be virally suppressed than women.^1^ Client barriers to care, such as missed wages, transport costs, and long waiting times for clinic visits and ART refills, are associated with detectable viral load.^6^, ^7^ These barriers are amplified among men who are less likely to seek care in part due to gender norms and stigma.^8^ Innovations in efficient service delivery—including expanding differentiated services to people with detectable viral load—are needed for people living with HIV, particularly men and people aged younger than 30 years, to achieve the UNAIDS 90-90-90 goal of 73% viral suppression among all people living with HIV.^1^, ^9^

**Research in context**

**Evidence before this study**

Although the proportion of people living with HIV who know their status has increased, approaching the UNAIDS goal of 90%, the proportion of people living with HIV who have started lifesaving antiretroviral therapy (ART) and met the gold-standard metric of success, viral suppression, lags behind by about a third, particularly among men. Viral suppression decreases morbidity and mortality for people living with HIV and stops transmission to their partners. Before this study, randomised trials had not tested the effectiveness of community-based ART initiation, monitoring, and resupply; an innovative approach to overcome barriers associated with accessing clinic-based services such as clinic hours and transport costs. Observational evidence suggests that community-based ART could increase ART initiation and viral suppression. A systematic review and meta-analysis from 2019 evaluated the effectiveness of community-based HIV initiatives in achieving the UNAIDS 90-90-90 targets; 90% of people living with HIV knowing their status, 90% of those starting ART, and 90% of those reaching viral suppression. In the meta-analysis, 11 databases were searched to identify community-based interventions for HIV published between 2007 and 2018. 37 studies were identified that evaluated community-based strategies to increase viral suppression. Success was defined as the intervention achieving 73% viral suppression (at the population level). For the outcome of viral suppression, community health workers and peers increased the relative risk of viral suppression by 40% (pooled OR 1·40, 95% CI 1·06–1·86) and the authors encouraged evaluation of community-based delivery of ART. Another study evaluated the effect of starting ART at the clinic and then transferring stable clients to decentralised medication delivery and adherence clubs (a hybrid clinic-community approach) on viral suppression compared with remaining at the clinic. They found no decrease in viral suppression with this clinic-community hybrid approach, showing the effectiveness of community ART refills. Test and treat studies for HIV prevention, which had a poor enrolment of men at 28% in KwaZulu-Natal, South Africa, and 29% in Zambia and South Africa, did not show the expected effect on HIV incidence, which might be due in part to not reaching enough men for HIV services. Comprehensive community-based services have the potential to close the gap in ART coverage for different demographics.

**Added value of this study**

This study presents new evidence from a randomised clinical trial in South Africa and Uganda on the effectiveness of community-based ART initiation, monitoring, and resupply to reach higher viral suppression, particularly among men. We present the intervention costs and a scenario in which the cost could be potentially similar or lower per person who is virally suppressed, compared with the current clinic approach.

**Implications of all the available evidence**

We showed that community-based initiation and delivery of ART is an effective strategy and could be scaled up to address the gap in viral suppression overall and for men in particular. Although this client-centred approach will require adaptation of services including expanding to new delivery platforms, cost might not be a limiting factor due to the increase in health gains seen with a high proportion of people reaching viral suppression.

Community-based ART services (ie, ART initiation and refills outside the clinic) have the potential to increase viral suppression by removing logistical barriers to clinic access and engaging people living with HIV in care.^10^, ^11^ Few data from randomised trials directly compare the efficacy, cost, and safety of comprehensive community-driven ART services, based entirely outside the clinic, to clinic services.

We did a randomised trial to evaluate the effectiveness of community-based ART initiation, monitoring, and resupply, and ART initiation at the clinic followed by monitoring and resupply in the community (hybrid approach), compared with standard clinic ART initiation, monitoring, and resupply for people with detectable HIV viral load in South Africa and Uganda. The primary objectives were to evaluate the relative effectiveness of community-based ART versus clinic-based ART on the proportion of people living with HIV who had viral suppression and the cost per person virally suppressed through community-based ART delivery.

## Methods

### Study design and participants

We did a multicentre, unblinded, household-randomised trial of community-based ART (ART initiation, monitoring, and resupply) and a hybrid approach (ART initiation at the clinic with community monitoring and resupply), compared with standard clinic ART delivery among people from South Africa and Uganda with detectable HIV viral load (>20 copies per mL; the lower limit of detection). We hypothesised that community-based ART would overcome logistical barriers to care, simplify monitoring and ART resupply, and increase viral suppression rates. Further, the increased cost of community-based ART delivery would be offset by the higher proportion of clients reaching viral suppression. Also, we hypothesised that starting ART at the clinic and then transferring monitoring and resupply to the community would cost less but the proportion virally suppressed would be lower than with community-based ART initiation. Finally, we hypothesised that community-based ART would increase viral suppression among men, who are less likely to link to HIV care at the clinic.

The DO ART study was done in rural and peri-urban areas of high and medium HIV prevalence in South Africa and Uganda: 11 communities in uMgungundlovu District, KwaZulu-Natal Midlands, South Africa; five communities in the uMkhanyakude District, northern KwaZulu-Natal, South Africa; and six in the Sheema District in southwest Uganda. Population HIV prevalence in KwaZulu-Natal was 36%,^12^ and in Mbarara, adjacent to the Sheema District was 11%,^13^ representing high and medium prevalence settings in southern and eastern Africa. All settings had public clinics that offered access to ART at no cost, and, since 2016, universal access to ART at all CD4 cell count measurements. These communities are characterised by high unemployment, low per-capita income (less than US$2 per day), and income inequality.

Following community mobilisation, participants were recruited largely through HIV testing at community locations and at home. Participants were also referred from clinics that did rapid HIV testing and community-based distribution of HIV self-test kits.

Trained nurses and supervised lay counsellors conducted study activities including ART initiation, refills, and monitoring. Staff received standardised national training in nurse-led HIV testing and counselling; clinical evaluation for ART initiation; ART initiation, monitoring, and adverse effects; and national algorithms for HIV care. Participants completed a questionnaire that included demographics and HIV risk with the mobile phone software Mobenzi Gateway (Cape Town, South Africa). Lay counsellors did HIV testing with standardised pre-test and post-test counselling and couples counselling, including mutual disclosure counselling. People living with HIV received additional point of care testing to stage their HIV and assess clinical eligibility for community-based ART initiation: CD4 cell count, WHO clinical HIV stage, pregnancy testing, creatinine testing to assess renal function, and symptom screening for tuberculosis. A dried blood spot was collected to assess HIV viral load at baseline.

People living with HIV were eligible for random assignment if they were able to provide informed consent, aged 18 years or older, resident in the participating communities, clinically stable (CD4 cell count >100 cells per μL, WHO HIV stage 1–3, not pregnant, normal renal function, and had no symptoms on a standardised symptom screen for active tuberculosis), and not taking ART at the time of assessment or in the past 3 months. 1 year after study activation, we added the inclusion criteria of detectable viral load because of a higher than expected proportion of participants with suppressed viral load at baseline, probably reflecting pre-existing diagnosis of HIV and undisclosed ART use as has been reported in other settings.^14^, ^15^ Participants with a suppressed viral load were retained in the study and completed all study procedures according to their study group. Participants who were not eligible for random assignment for clinical reasons or pregnancy were referred to care and followed-up until they were linked to clinic-based services. We chose a CD4 threshold of more than 100 cells per μL to facilitate clinic care for people with advanced HIV or AIDS who are at risk for opportunistic infections.

Before enrolment, all participants provided written informed consent, which included counselling about randomisation, procedures in each study group, and their rights as research participants. The Human Sciences Research Council Research Ethics Committee in South Africa, the Mbarara University of Science and Technology Research Ethics Committee in Uganda, and the University of Washington Institutional Review Board, Seattle, WA, USA approved the study.

### Randomisation and masking

The study biostatistician generated the randomisation allocation stratified by site and country. The randomisation allocation was automatically assigned by mobile phone software to the study participant. Once eligibility was assessed, the randomisation assignment was revealed to the participant and staff. The study staff did not have access to the randomisation code. Eligible participants in the same household were randomly assigned to the same group to prevent crossover between study groups. Individuals living with HIV with detectable viral load were randomly assigned (1:1:1) to the community, hybrid, or clinic groups (figure). Because of the difficulty in masking the study team and study participants to the ART delivery method, the study was unblinded; however, the laboratory staff, who assessed the primary outcome of plasma HIV viral load, were masked to the allocation of participants, as were the study investigators.

**Figure:**
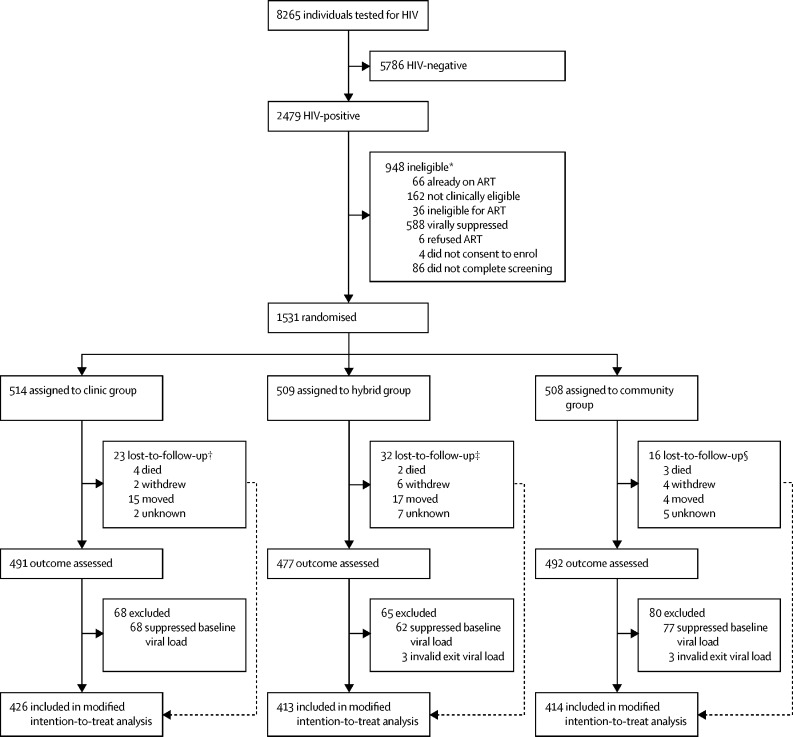
Trial profile ART=antiretroviral therapy. *Participants were counted once for the first exclusion criteria they met. †Three participants lost-to-follow-up contributed to the modified intention-to-treat analysis with viral loads ascertained from their clinic charts. ‡One participant lost-to-follow-up contributed to the modified intention-to-treat analysis with viral loads ascertained from their clinic charts. §Two participants lost-to-follow-up contributed to the modified intention-to-treat analysis with viral loads ascertained from their clinic charts.

### Procedures

Participants in the community-based ART delivery group received same-day ART initiation including standardised counselling and the national HIV programmes' first-line efavirenz-based ART regimen. 7 days after ART initiation, participants received a telephone call to ask about symptoms, ART side-effects, and adverse events. After enrolment, participants returned for in-person follow-up visits at month 1, 3, 6, 9, and 12 for ART resupply, clinical monitoring, counselling, and ascertainment of adverse events and social harms. Follow-up visits were done at a mobile van parked at a known location and at a scheduled time. ART supply was dispensed for 1 month, 2 months, and then every 3 months thereafter. Trimethoprim-sulfamethoxazole prophylaxis was dispensed according to country guidelines and tuberculosis preventive therapy (isoniazid) was provided from month 1. Participants received appointments for their mobile visits, with an automated text message reminder the week before their visit. Participants were able to reschedule visits by text message, request additional ART resupply if travelling, and nominate someone else to collect their medication. Participants who missed visits were contacted and their visit rescheduled. The mobile service was regularly available on evenings and weekends. Staff used a mobile phone application for standardised monitoring that included counselling guidelines and required all the steps to be completed before the encounter was closed. For HIV and ART monitoring, participants completed a clinical questionnaire to screen for symptoms of ART adverse events, tuberculosis, and other common opportunistic infections. Point-of-care creatinine testing was done to monitor renal function. HIV plasma viral load was assessed for treatment success at month 6. Text messages were sent with viral load results without mentioning HIV to protect confidentiality regarding their HIV infection and next steps according to whether they had detectable or undetectable viral load (eg, “All is going well. Keep up the good work.” or “Please contact us for more information.”). Participants received individualised adherence support. Participants who required additional clinical services were referred for care and followed up until they were linked to a clinic. Participants in the community-based ART delivery group were administratively linked to the clinic and their files kept up to date.

Participants in the hybrid group were counselled to notify the study team once they had initiated ART at the clinic to facilitate transition to the community for refills and monitoring. Until notification of ART initiation, they received quarterly telephone calls to enquire as to whether they had initiated ART and to record adverse events. Once participants had initiated ART at the clinic, they had community-based monitoring and refills, following identical procedures for the community-based ART delivery group and thus described as a hybrid between the clinic and community-based strategies.

Participants in the clinic group were referred to established local ART clinics for ART initiation, monitoring, and refills. They received quarterly telephone calls to document ART initiation and adverse events.

Social harms and adverse events were assessed at every in-person visit and with every telephone call. Participants were asked about adverse events, including serious adverse events; we included admission to hospital related to HIV and ART among serious adverse events. Chart abstraction was done for all participants to capture additional clinical events and test results.

At the exit visit 12 months after random assignment, all participants completed an in-person visit comprising the follow-up procedures for the community-based ART delivery group as outlined above, including phlebotomy for measurement of HIV viral load in plasma. In addition, all participants completed a questionnaire regarding their experience in accessing care, acceptability of community-based ART, and barriers for not visiting the clinic in the clinic group. Clinic chart abstraction was done for all study participants to verify ART initiation and refills and identify adverse events not already reported. Participants receiving community-based ART were then transferred to the clinic or decentralised medication dispensing as appropriate.

### Outcomes

The primary trial endpoint was the proportion of participants who had HIV viral suppression (<20 copies per mL) assessed at month 12; plasma viral load testing was done by an accredited laboratory at each site. The cost per person who was virally suppressed in the community-based group was a coprimary endpoint assessed through activity-based microcosting. Safety was a secondary outcome and was assessed through adverse event reporting and clinical chart abstraction. Viral suppression in South Africa and among men were prespecified secondary outcomes.

Rapid HIV testing was done according to national guidelines: in Uganda, Determine HIV 1–2 (Abbott Laboratories, Abbott Park, IL, USA) followed by Statpak (Chembio Diagnostics, Medford, NY, USA) for confirmation of positive results, and Unigold (Trinity Biotech, Bray, Ireland) was used if results were not confirmatory. In South Africa, Determine HIV 1–2 (Abbott Laboratories) and First Response HIV 1–2-0 Card Test (Prima Medical, Kachigam, India) with SD BIOLINE HIV-1/2 Rapid (G-Ocean, Hong Kong) were used if results were not confirmatory. Creatinine testing was done with point-of-care StatSensor Xpress (Nova Biomedical, Waltham, MA, USA) in South Africa or via a laboratory based COBAS INTEGRA 400 (Roche, Mannheim, Germany) platform in Uganda. Point-of-care CD4 cell count testing (Pima, Alere, Waltham, MA, USA) was done using a finger-stick specimen. Plasma HIV viral load was assessed by PCR (COBAS AmpliPrep or TaqMan, Roche, Mannheim, Germany, with a limit of detection of 20 copies per mL) in Uganda and bDNA (bioMérieux, Marcy-l'Étoile, France, with a limit of detection of 20 copies per mL) in South Africa. Baseline viral load was tested from dried blood spots via bDNA (bioMérieux, with a limit of detection of 20 copies per mL).

We did activity-based microcosting to assess the cost of community-based ART, including time and motion studies to estimate the number of clients that could be seen in 1 day. We estimated the annual per-client cost of community-based ART care from the provider's perspective. We report separate cost estimates for the first year of ART (including start-up costs, ART initiation, and five follow-up visits) and additional years of ART (consisting of four follow-up visits once every 3 months). Costs included all activities and supplies supporting ART initiation and follow-up including personnel, laboratory testing, and medications (appendix pp 12–13). We annualised start-up, vehicle, and equipment costs over duration of use of the items (ie, useful life) with a discount rate of 3%.^16^ The costs are presented in 2018 US dollars (US$), as 2018 was the midpoint of the study. To reflect implementation under a scaled-up scenario, we estimated costs with South African and Ugandan Ministry of Health salaries and projected client volume using the time and motion data. We compared scaled-up costs of community ART delivery with estimates from previous studies of the annual per-person estimates of the cost of clinic ART in South Africa^17^ and Uganda.^18^ We used the study viral suppression rates and costs to estimate the cost per person virally suppressed through community-based ART compared with clinic ART (appendix p 13).

### Statistical analysis

A target sample size of 1200 individuals living with HIV not on ART was calculated to provide at least 90% power to see a 10% absolute difference in viral suppression (estimated 65% viral suppression in the clinic group, 75% in the hybrid group, and 85% in the community-based ART delivery group), assuming 5% loss to follow-up and a significance level of 0·05. Because of the unexpectedly high number of participants with suppressed viral loads at baseline (who we planned to exclude from the modified intention-to-treat primary analysis) and to sufficiently power the study for a subgroup analysis among men, the protocol was modified to allow enrolment of up to 1800 participants, with a target of at least 600 men with detectable baseline viral load. We calculated that with 600 men, we would have more than 90% power to see a 15% change in viral load suppression (45% in the clinic group, 60% in the hybrid group, and 75% in the community group). All available month 12 assessments from participants contributed to assignment of whether the participant was virally suppressed.

Effects of the randomisation groups on viral suppression were estimated as relative risks (RRs) produced with Poisson regression generalised estimating equations with robust variance estimation that accounted for within-household correlation.^19^ Models were adjusted a priori for gender, age less than 30 years, baseline CD4 cell count category, and study site, which are known covariates of viral suppression. Tests for superiority of the two intervention groups compared with the clinic group were based on two-sided Wald p values less than 0·05. In the absence of significant evidence for superiority, an a-priori test for non-inferiority was done to test for a RR of more than 0·95 based on a one-sided Wald p value of less than 0·025. Effect modification was evaluated with interaction terms for gender, age, and site.

The primary analyses were by modified intention-to-treat, excluding participants who were virally suppressed at baseline. We did sensitivity analyses on the overall model to test our assumptions, such as an analysis by intention-to-treat, including those who were virally suppressed at baseline. Additional sensitivity analyses used the WHO cutoff for viral suppression of less than 1000 copies per mL and adjusted for time. We did all analyses with R (version 3.6).

An independent data safety and monitoring board met approximately annually during the study to review study progress and participant safety. This study was registered with ClinicalTrials.gov, NCT02929992.

### Role of the funding source

The funders of the study had no separate role, beyond that of other authors, in study design, data collection, data analysis, data interpretation, or writing of the report. The corresponding author had full access to all the data in the study and had final responsibility for the decision to submit for publication.

## Results

Between May 26, 2016, and March 28, 2019, 9094 participants were identified for HIV testing and counselling and 8265 (90·9%) were tested (figure). Of those tested, 3957 (47·8%) were men. Overall, 2479 (30·0%) participants tested positive for HIV and 2393 (96·5%) of the participants who tested positive completed screening for the DO ART Study. Of the 862 individuals who were ineligible for randomisation, 588 (68%) were virally suppressed at baseline, 66 (8%) reported currently being on ART, 76 (9%) screened positive with the symptomatic TB screening questionnaire, 36 (4%) had a CD4 cell count of less than 100 cells per μL, and 26 (3%) were pregnant. 1531 participants were randomly assigned: 514 (34%) to the clinic group, 509 (33%) to the hybrid group, and 508 (33%) to the community-based group. 71 participants were lost to follow-up: 23 (32%) in the clinic group, 32 (45%) in the hybrid group, and 16 (23%) in the community-based group; of the 71, nine (13%) died, 12 (17%) withdrew, 36 (50%) moved, and 14 (20%) were lost to follow-up for an unknown reason. At least 417 (82%) participants completed each visit (months 1, 3, 6, and 9) in the community-based ART delivery group (appendix p 7). 1466 (95·8%) of 1531 contributed a viral load endpoint to an analysis. We planned a priori to exclude participants with suppressed baseline viral load (n=216) for the modified intention-to-treat primary analysis, which meant 1315 were included in the modified intention-to-treat analysis: 446 in the clinic group, 442 in the hybrid group, and 427 in the community group. Of the 1315 participants in the analysis, the primary outcome of HIV viral load was available for 1253 (95·3%).

The baseline characteristics for the 1315 participants in the primary modified intention-to-treat analysis are shown in table 1, of whom 666 (51%) were men. Participants had a median age of 32 years (IQR 27–40). 879 (69%) of 1282 participants completed secondary education and 772 (59%) of 1315 reported that they were not employed. 989 (76%) of 1305 participants reported one current sex partner, 295 (23%) of 1296 reported condom use at last sexual intercourse, and 144 (22%) of 666 men were circumcised. Most participants were asymptomatic and clinically stable: 1176 (89%) of 1315 were WHO clinical stage I, 862 (66%) of 1315 had a CD4 cell count of 350 cells per μL or more, and 1243 (95%) of 1315 had normal renal function (creatinine <106 μmol/L).

**Table 1:** Baseline characteristics

		**Clinic group (n=446)**	**Hybrid group (n=442)**	**Community group (n=427)**	**Total (N=1315)**
Gender					
	Men	230 (52%)	217 (49%)	219 (51%)	666 (51%)
	Women	216 (48%)	225 (51%)	208 (49%)	649 (49%)
Age, years					
	18–29	150 (34%)	179 (40%)	158 (37%)	487 (37%)
	30–49	264 (59%)	230 (52%)	234 (55%)	728 (55%)
	≥50	32 (7%)	33 (7%)	35 (8%)	100 (8%)
Education					
	Primary	136/439 (31%)	144/431 (33%)	123/412 (30%)	403/1282 (31%)
	Secondary	289/439 (66%)	275/431 (64%)	272/412 (66%)	836/1282 (65%)
	Tertiary	14/439 (3%)	12/431 (3%)	17/412 (4%)	43/1282 (3%)
Employed		177 (40%)	190 (43%)	176 (41%)	543 (41%)
Study household size					
	1	425 (95%)	424 (96%)	407 (95%)	1256 (96%)
	2	21 (5%)	18 (4%)	20 (5%)	59 (4%)
In relationship		276 (62%)	285 (64%)	279 (65%)	840 (64%)
Number of current sexual partners					
	0	37/440 (8%)	40/439 (9%)	34/426 (8%)	111/1305 (9%)
	1	331/440 (75%)	332/439 (76%)	326/426 (77%)	989/1305 (76%)
	≥2	72/440 (16%)	67/439 (15%)	66/426 (15%)	205/1305 (16%)
Condom used at last sex		108/439 (25%)	94/435 (22%)	93/422 (22%)	295/1296 (23%)
Circumcised if male		54/230 (23%)	43/217 (20%)	47/219 (21%)	144/666 (22%)
Patient aware of nearby HIV clinic		436 (98%)	436/441 (99%)	415 (97%)	1287/1314 (98%)
WHO stage					
	Stage 1	395 (89%)	399 (90%)	382 (89%)	1176 (89%)
	Stage 2	40 (9%)	36 (8%)	40 (9%)	116 (9%)
	Stage 3	11 (2%)	7 (2%)	5 (1%)	23 (2%)
CD4 cell count, cells per μL					
	100–349	150 (34%)	143 (32%)	160 (37%)	453 (34%)
	350–499	106 (24%)	107 (24%)	110 (26%)	323 (25%)
	≥500	190 (43%)	192 (43%)	157 (37%)	539 (41%)
Creatinine, μmol/L					
	<106	418 (94%)	417 (94%)	408 (96%)	1243 (95%)
	106–133	28 (6%)	25 (6%)	19 (4%)	72 (5%)
Dried blood spot viral load, copies per mL					
	20–999	89/404 (22%)	96/404 (24%)	79/396 (20%)	264/1204 (22%)
	1000–9999	156/404 (39%)	145/404 (36%)	175/396 (44%)	476/1204 (40%)
	≥10 000	159/404 (39%)	163/404 (40%)	142/396 (36%)	464/1204 (39%)

Data are n (%). Data are for the modified intention-to-treat population.

Overall, community-based ART increased viral suppression compared with the clinic group (74% *vs* 63%, RR 1·18, 95% CI 1·07–1·29; p_superiority_=0·0005) and the hybrid approach was non-inferior to the clinic group (68% *vs* 63%, RR 1·08, 0·98–1·19; p_non-inferiority_=0·0049; RR >0·95; table 2). Both the community and hybrid strategies significantly increased viral suppression among men: community-based ART (73%, RR 1·34, 95% CI 1·16–1·55; p_superiority_<0·0001) and the hybrid approach (66%, RR 1·19, 1·02–1·40; p_superiority_=0·026), compared with standard of care (54%). Notably, there was a significant interaction between gender and study group for both the community-based ART (p=0·0067) and the hybrid group (p=0·035) compared with clinic ART. Viral suppression was similar for men (156 [73%] of 213) and women (150 [75%] of 201) in the community-based ART group, compared with 120 (54%) of 221 for men and 149 (73%) of 205 for women in the clinic group. Overall, the absolute increase in viral suppression was 18·4% (95% CI 9·5–27·2) for men in the community-based group compared with the clinic-based group (appendix p 9).

**Table 2:** Rates and relative risks of viral suppression

		**Rate of viral suppression**			Adjusted*RR of viral suppression			
		Clinic group	Hybrid group	Community group	Hybrid group versus clinic group		Community group versus clinic group	
					RR (95% CI)	p value	RR (95% CI)	p value
Overall for South Africa and Uganda		269/426 (63%)	282/413 (68%)	306/414 (74%)	1·08 (0·98–1·19)	0·12; 0·0049†	1·18 (1·07–1·29)	0·0005
Gender overall		..	..	..	..	0·035‡	..	0·0067‡
	Men	120/221 (54%)	134/203 (66%)	156/213 (73%)	1·19 (1·02–1·40)	..	1·34 (1·16–1·55)	..
	Women	149/205 (73%)	148/210 (70%)	150/201 (75%)	0·98 (0·87–1·10)	..	1·04 (0·93–1·17)	..
Age overall, years		..	..	..	..	0·85‡	..	0·50‡
	18–29	98/144 (68%)	114/160 (71%)	116/154 (75%)	1·07 (0·92–1·24)	..	1·14 (0·98–1·31)	..
	≥30	171/282 (61%)	168/253 (66%)	190/260 (73%)	1·08 (0·96–1·23)	..	1·20 (1·07–1·35)	..
Site		..	..	..	..	0·21‡	..	0·16‡
	Southwestern Uganda	64/84 (76%)	66/91 (73%)	74/94 (79%)	0·97 (0·81–1·16)	..	1·06 (0·90–1·24)	..
	Midlands KwaZulu-Natal, South Africa	125/213 (59%)	137/216 (63%)	151/220 (69%)	1·08 (0·93–1·25)	..	1·17 (1·01–1·35)	..
	Northern KwaZulu-Natal, South Africa	80/129 (62%)	79/106 (75%)	81/100 (81%)	1·20 (1·01–1·42)	..	1·31 (1·11–1·55)	..
South Africa		205/342 (60%)	216/322 (67%)	232/320 (72%)	1·12 (1·00–1·25)	0·055; 0·0026†	1·22 (1·09–1·36)	0·0004
Gender for South Africa		..	..	..	..	0·037‡	..	0·015‡
	Men	91/178 (51%)	100/153 (65%)	113/158 (72%)	1·26 (1·04–1·51)	..	1·39 (1·17–1·66)	..
	Women	114/164 (70%)	116/169 (69%)	119/162 (73%)	0·99 (0·86–1·15)	..	1·07 (0·93–1·22)	..

Data are n (%) unless specified. Data are for the modified intention-to-treat population, overall and among subgroups. RR=relative risk.

* Adjusted for gender, age younger than 30 years, baseline CD4 cell count (WHO category), and study site.

† p value for a one-sided Wald test for non-inferiority (RR >0·95).

‡ p value for a Wald test for significant interaction.

In a pre-planned subgroup analysis for South Africa, compared with clinic ART, community-based ART increased viral suppression (60% *vs* 72%, RR 1·22, 95% CI 1·09–1·36) and the hybrid approach was non-inferior (60% *vs* 67%%, RR 1·12, 1·00–1·25; p_non-inferiority_=0·0026, RR >0·95). In South Africa, both community strategies significantly increased viral suppression among men: community-based ART (72%, RR 1·39, 95% CI 1·17–1·66) and the hybrid approach (65%, RR 1·26, 1·04–1·51), compared with standard of care (51%). There was significant interaction for gender and study group for both community-based ART (p=0·015) and the hybrid group (p=0·037) compared with clinic ART in the South African subset. The absolute increase in viral suppression was 20·4% (95% CI 10·0–30·3) for South African men in the community-based group compared with the clinic-based group (appendix p 9).

We did multiple sensitivity analyses to evaluate the effect of our assumptions on the study outcomes (appendix p 8). Specifically, we included participants who were virally suppressed at baseline; the intention-to-treat analysis. This method did not change the primary outcome that, compared with clinic-based ART, community-based ART was superior (65% *vs* 75%, RR 1·15, 95% CI 1·06–1·25) and the hybrid approach remained non-inferior (65% *vs* 69%, RR 1·06, 0·98–1·16; p_non-inferiority_=0·0054, RR>0·95). In further pre-planned sensitivity analyses, including using the WHO viral suppression threshold of less than 1000 copies per mL, community-based ART remained superior overall, and the hybrid group was non-inferior versus clinic ART. In a post-hoc analysis, participants enrolled after the median site enrolment date were not more likely to have viral suppression (p=0·81).

Assuming that 7–13 clients are seen per work day, and including the cost of ART, trimethoprim-sulfamethoxazole, laboratory testing, personnel, supplies, fuel, and overheads, we estimated that the annual cost of community-based ART per client was $217 in Uganda and $308–312 in South Africa in the first year and $187 in Uganda and $244–246 in South Africa in subsequent years (table 3). The cost of ART ranged from $102–114 in South Africa and Uganda. Using the annual cost of $249 per person for clinic-based ART^17^ in South Africa and the viral suppression rates observed in the study groups, the annual cost per person virally suppressed was $402–422 in the clinic group and $325–390 in the community-based group in South Africa (appendix p 13). Similarly in Uganda, using the annual cost of $291 per person for streamlined clinic-based ART,^18^ the annual cost per person virally suppressed was $214 in the clinic-based group and $275 in the community-based group.

**Table 3:** Annual cost per client of community-based ART

	**First year costs**			**Subsequent year costs**		
	Southwestern Uganda	Midlands KwaZulu-Natal, South Africa	Northern KwaZulu-Natal, South Africa	Southwestern Uganda	Midlands KwaZulu-Natal, South Africa	Northern KwaZulu-Natal, South Africa
Drugs	$110 (51%)	$118 (38%)	$118 (38%)	$110 (59%)	$118 (48%)	$118 (49%)
Personnel	$23 (11%)	$99 (32%)	$90 (29%)	$19 (10%)	$69 (28%)	$64 (26%)
Laboratories	$36 (16%)	$60 (19%)	$60 (19%)	$22 (12%)	$34 (14%)	$34 (14%)
Vehicles	$11 (5%)	$9 (3%)	$12 (4%)	$9 (5%)	$6 (3%)	$8 (3%)
Start-up	$3 (1%)	$11 (3%)	$12 (4%)	$2 (1%)	$8 (3%)	$9 (4%)
Fuel	$17 (8%)	$4 (1%)	$4 (1%)	$13 (7%)	$3 (1%)	$3 (1%)
Building	$10 (5%)	$8 (3%)	$7 (2%)	$8 (4%)	$6 (2%)	$5 (2%)
Equipment	$5 (2%)	$2 (1%)	$4 (1%)	$1 (<1%)	<$1 (<1%)	<$1 (<1%)
Other	$2 (1%)	$1 (<1%)	$1 (<1%)	$2 (1%)	$1 (<1%)	$1 (<1%)
Total	$217 (100%)	$312 (100%)	$308 (100%)	$187 (100%)	$246 (100%)	$244 (100%)

Data are cost (% of total cost). Cost is in 2018 US$.

Serious adverse events occurred in 20 (1%) participants: eight in the clinic group, five in the hybrid group, and seven in the community group (all 1%; appendix p 10); 14 (70%) of 20 of the serious adverse events (seven in the clinic group, one in the hybrid group, and six in the community group) were considered related or possibly related to HIV. Excluding serious adverse events, 13 (1%) participants had a severe adverse event (all grade 3) in the study: two in the clinic group, four in the hybrid group, and seven in the community group, of which nine were due to high blood pressure, which was measured routinely in the community groups and once in the clinic group. Excluding the high blood pressure readings, of the four participants with other severe adverse events, one occurred in the clinic group, two in the hybrid group, and one in the community-based group. Reported social harms related to trial participation occurred in two participants; both participants were in the community-based group.

## Discussion

This randomised trial in high and medium HIV prevalence settings in South Africa and Uganda provides evidence that community-based delivery of ART, including HIV testing, same-day ART initiation, mobile van monitoring, and ART resupply increases viral suppression among people living with HIV with detectable viral load, particularly among men, compared with standard clinic-based services. The hybrid approach of ART initiation at the clinic, followed by monitoring and refills in the community, was non-inferior than clinic ART suggesting that streamlined services could be beneficial from the first step of testing and throughout the continuum of HIV care for people not already in care. Streamlined services that overcome barriers to care can increase the proportion of people living with HIV who start ART and reach viral suppression.

We hypothesised that community-based HIV testing, ART initiation, monitoring, and resupply would increase the proportion of individuals living with HIV who reach viral suppression. This theory was based on observational evidence that community health-care worker-led HIV interventions increased suppression.^20^ Barriers to care include standard opening hours, stigma, unfavourable perceptions of clinics and staff, cost of transport, and lost wages.^7^, ^21^, ^22^ Men might experience additional barriers such as gender norms that inhibit men from seeking care.^8^ As a result, many individuals do not initiate ART at conventional facilities.^8^, ^23^ After ART initiation, guidelines might require that clients show retention in care for 12 months and viral suppression before accessing decentralised ART refills.^5^ Requiring multiple clinic visits contributes to loss to follow-up over the first year of ART.^24^ If clients are transferred to ART refills outside the clinic (eg, decentralised dispensing, adherence groups, community, and home) compared with clinic ART, they remained virally supressed.^25^, ^26^, ^27^ Offering convenient locations and times after hours and on weekends, being flexible to meet travel or mobility needs, offering quarterly refills, and streamlining monitoring and resupply had better viral suppression outcomes, especially for men.

In pre-planned subgroup analyses, we found that the difference between the community-based group and the clinic-based group was higher for men, and this difference was present in the overall sample and when restricted to data from South Africa, where both the community and hybrid approaches significantly increased viral suppression for men. Notably, the UNAIDS 90-90-90 goal of 73% viral suppression among people living with HIV (in this case, with detectable viral load) was met for both men and women in the community-based ART group, eliminating disparities by gender. Universal test and treat HIV prevention studies have shown either no difference or a smaller than expected decrease in HIV incidence at the population level,^28^, ^29^ possibly due in part to the low proportion of men enrolled (<30%). In a generalised HIV epidemic setting, high coverage of viral suppression among women and men could lower incidence.^30^, ^31^ The study was not powered to estimate the effect of community-based ART among women only.

At scale, we project that the cost of community-based ART could be similar to previously published estimates of the cost of clinic ART.^17^, ^18^ However, questions remain regarding how community-based ART could be implemented at scale in the absence of established delivery platforms and systems. Refining components of the community-based ART delivery strategy could decrease or contain costs, which might be necessary in areas where the density of clients is low. For example, providing 6-month resupply of ART instead of 3-month resupply could save personnel costs with no difference in viral suppression or retention in care.^32^ Alternative methods to increase viral suppression while containing costs also warrant comparison.^33^, ^34^

Although community-based approaches to ART delivery have growing policy support, implementation is at an early stage. Concerns raised about community-based ART include the safety of clients who would not have regular contact with the clinic and that streamlined care could lead to less engagement in care. We found similar rates of serious and severe adverse events across the three study groups, indicating that community-based ART is likely to be as safe as clinic-based ART. Further, the proportion of people reaching viral suppression was higher through community-based ART, which could increase health gains over time. Lastly, for scale-up, implementation will need a mix of behavioural and social interventions to see the same (or larger) effect sizes across heterogeneous settings.

The study has several limitations. More than half of the participants succeeded at the clinic and it is probable that additional changes to the standard of care at the clinic (eg, fast-track ART, which was available later in the study) could have improved viral suppression in the clinic-based group, attenuating the effect. However, the date of enrolment did not change the primary finding for viral suppression. Importantly, missing data for viral load at exit among participants lost to follow-up did not change the primary result (appendix p 8). We used a literature estimate as a proxy for clinic-based costs. The comparison with the clinic costs was not generated from the clinics our participants attended. However, a target cost of $200 per client on ART per year (including ART, clinical care, and laboratory monitoring) is used for the investment case for HIV treatment.^35^ We present the primary costing results herein; a planned cost-effectiveness analysis will be done to evaluate the health gains associated with the change in costs. Our study was limited to settings with medium and high HIV prevalence and might not be generalisable to settings with lower prevalence, as sufficient volume is required for community-based ART to be cost-efficient. The study was limited to adults because efficacy and safety of community-based ART had not been established in people younger than 18 years. Adolescents are a priority group and should be included in future evaluations of community-based ART.

The strengths of the study include the randomised design, the use of a clinical mobile app, successful enrolment of men (51% of the study population; previous studies have typically enrolled <30% men), and high retention across all three randomised groups. The study's coprimary aims were effectiveness, measured by proportion of participants who were virally suppressed, and cost per person virally suppressed so that affordability of community-based ART delivery could be considered in addition to effectiveness. With use of a mobile app, standardised care was provided following clinical algorithms thus limiting medical errors and facilitating task shifting.

The next steps for community-based ART are testing at scale and evaluating the costs and effect in other high HIV prevalence settings with a baseline gender gap in suppression. Although community-based ART resulted in viral suppression among approximately three-quarters of individuals living with HIV, additional services are needed to reach the remaining quarter. Specifically, home delivery of ART and long-acting injectable ART could overcome remaining logistical barriers. Future research directions should focus on scalable, client-centred strategies to deliver ART.

## Data sharing

A complete de-identified patient dataset sufficient to reproduce the study findings will be made available approximately 1 year after completion of the trial (January, 2021), following approval of a concept sheet summarising the analyses to be done. Further inquiries can be directed to the DO ART Scientific Committee at rbarnaba@uw.edu.
