# Supplementary material for: Community-based antiretroviral therapy versus standard clinic-based services for HIV in South Africa and Uganda (DO ART): a randomised trial
Source: Lancet Glob Health. 2020 Sep 21;8(10):e1305–15. doi: 10.1016/S2214-109X(20)30313-2 (PMC7527697; doi:10.1016/S2214-109X(20)30313-2)
Supplement: Supplementary appendix [file mmc1.pdf]

THE LANCET
Global Health

Supplementary appendix

This appendix formed part of the original submission and has been peer reviewed. We post it as supplied by the authors.

Supplement to: Barnabas RV, Szpiro AA, van Rooyen H, et al. Community-based antiretroviral therapy versus standard clinic-based services for HIV in South Africa and Uganda (DO ART): a randomised trial. *Lancet Glob Health* 2020; **8:** e1305–15.

DO ART STUDY
SUPPLEMENTARY MATERIAL

TABLE OF CONTENTS

DO ART Study Team ---- 3
Coordinating partners ---- 3
Study sites ---- 3
Independent ---- 3
Screening and Enrolment ---- 4
Figure: Participant accrual ---- 5
Table S1: Screening and enrolment ---- 5
Table S2: Baseline characteristics: intention-to-treat ---- 6
Retention ---- 7
Table S3: Study retention by timepoint ---- 7
Endpoints ---- 8
Table S4: Sensitivity analyses of relative risks of viral suppression, overall ---- 8
Table S5: Rates and risk differences of viral suppression at exit by group, overall and among subgroups ---- 9
Table S6: Rates and relatives risks of viral suppression at exit by group and gender ---- 10
Table S7: Severe adverse events and social harms – participant-level summary by group ---- 10
Cost Analysis Details ---- 11
Cost Estimation ---- 11
Scenarios ---- 11
Table S8: Estimated client volume by site for efficient at-scale scenario ---- 11
Table S9: Estimated number of clients seen per year per under steady-state and efficient at-scale scenarios ---- 12
Table S10: Unit costs of key inputs (2018 USD) ---- 12
*Obtained from study budgets ---- 12
Annual cost per client ---- 12
Table S11: Annual cost per client in steady-state (as observed) scenario (2018 USD) ---- 12
Table S12: Annual cost per client in steady-state (programmatic) scenario (2018 USD) ---- 12
Table S13: Annual cost per client in efficient at-scale scenario (2018 USD) ---- 13
Table S14: Estimated annual costs per client and per client virally suppressed under community-based ART delivery (2018 USD) 13
Table S15: Comparison of estimated community-based (efficient at-scale scenario) vs. facility-based annual cost per client virally suppressed in South Africa and Uganda ---- 13
Limitations ---- 13
Evolving Standard of Care ---- 14
Table S16. Summary of changes in standard of care by site ---- 14
Table S17. Standard of care description at Kabwohe Clinical Research Centre (SW Uganda) ---- 14
Table S18. Standard of care description at Caluza, Mpumuza, and Pata clinics (Midlands KZN SA) ---- 15
Table S19. Standard of care description at Ashdown clinic (Midlands KZN SA) ---- 15
Table S20. Standard of care description at Nkundusi, Madwaleni, Ntondweni, and Zwenelisha clinics (Northern KZN SA) ---- 15
Table S21. Standard of care description at Sipho Zungu clinic (Northern KZN SA) ---- 15

DO ART STUDY TEAM

Coordinating partners

University of Washington: Ruanne V. Barnabas, Connie Celum (principal investigators); Jared M. Baeten, Jennifer Baugh, Ellie Hawman, Jack Knauer, Meighan L. Krows, Rachel Johnson, Toni Maddox, Deidra Montoya, Susan Morrison, Colin Pappajohn, Kathryn Peebles, Justice Quame-Amaglo, D. Allen Roberts, Adrienne E. Shapiro, Torin T. Schaafsma, Adam Szpiro, Katherine K. Thomas

Harvard University: Norma Ware (principle investigator); Emily Pisarski, Monique Wyatt

Study sites

Integrated Community Based Initiatives (Southwestern Uganda): Stephen Asiimwe, Elioda Tumwesigye (principal investigators); Silvanus Ahaisibwe, Edwin Ariguma, Paul Asiimwe, Robert Biajuka, Mackline Birungi, Obed Masiko, Benard Muhumuza, Saidi Mureme, Julius Nabasa, Alison Nagasha, Mishaki Ndebesa, Edgar Ngarame Nduho, Caroline Orishaba, Moses Tukwatsibwe, John Bosco Tumuhairwe, Aggrey Tumuhimbise, Betty Tumukunde, Bosco Turyamureeba

Human Sciences Research Council (Mid-KZN SA): Alastair van Heerden, Heidi van Rooyen (principal investigators); Nomfundo Bhengu, Mbongeleni Buthelezi, Pumla Dladla, Beauty Dlamini, Philip Joseph, Nosipho Khumalo, Nonkululeko Khuzwayo, Celokuhle Latha, Lungelo Madlala, Sanelisiwe Majola, Langelihle Makhoba, Smiso Mbambo, Mbongeni Mbanjwa, Yvonne Mdakane, Tembeka Mhlakwaphalwa, Noneka Mhlaluka, Philisiwe Mkhize, Nomusa Mntambo, Jabu Molefe, Lindani Msimango, Thulani Msomi, Zilungile Mvubu, Protus Mzolo, Nontobeko Ndebele, Sabelo Ndlovu, Nkosinathi Ngcobo, Mbongeni Ngcoya, Thulani Ngubane, Siyabonga Nkala, Jabulile Ntombela, Njabulo Ntusi, Thembelihle Pita, Nhlanhla Radebe, Nokhanya Radebe, Princess Ralenkoane, Kombi Sausi, Wonderboy Shange, Kwenzakwakhe Shezi, Sibongile Shezi, Khayelihle Zondi, Nomvula Zulu, Sindiswa Zulu, Nobuhle Zuma

Africa Health Research Institute (Northern KZN SA): Olivier Koole, Deenan Pillay, Maryam Shahmanesh (principal investigators); Oluwafemi Adeagbo, Zilethile Khumalo, Tumi Madolo, Thuli Mahlaba, Sbusiso Mdletshe, Zodwa Mkhwanazi, Lethiwe Mlambo, Mthokozisi Mnomiya, Nondumiso Mpanza, Senzo Msweli, Sanele Mthiyane, Thabani Mtshali, Xolani Ngwenya, Nokwazi Ntombela, Siyabonga Nxumalo, Vuyiswa Nyawose, Janet Seeley, Cabangeni Shange, Funani Shange, Nsika Sithole

Independent

Mobenzi: Peter Fowles, Andi Friedman, Norval Geldenhuys, Lee Goodrick, Mark Katzwinkel, Elsje Kemp, Simone Leukes, Luckmore Muzarabani, Catherine Rode, Marinthea van Tonder, Ruaan Viljoen

Data and Safety Monitoring Board: Francois Venter (Chair), James P. Hughes, Sydney B. Rosen

SCREENING AND ENROLMENT

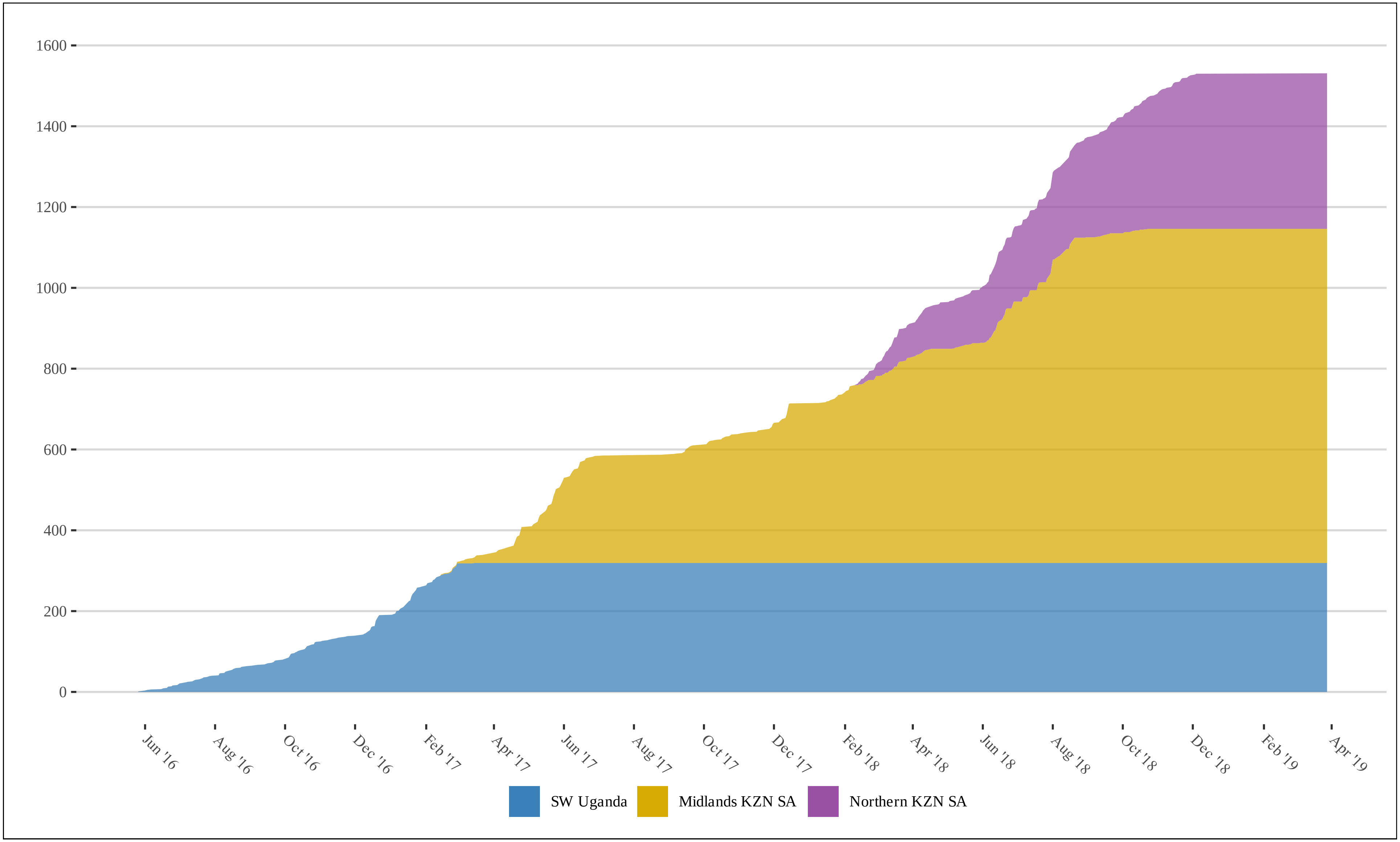

Figure: Participant accrual

Table S1: Screening and enrolment

	Total			SW Uganda			Midlands KZN SA			Northern KZN SA*		
	**Total**	**Male**	**Female**	**Total**	**Male**	**Female**	**Total**	**Male**	**Female**	**Total**	**Male**	**Female**
Enumerated	9094	4377	4717	2001	994	1007	6355	2993	3362			
Tested for HIV	8265 (91%)	3957 (90%)	4308 (91%)	1750 (87%)	856 (86%)	894 (89%)	5967 (94%)	2811 (94%)	3156 (94%)			
HIV+	2479 (30%)	1035 (26%)	1444 (34%)	398 (23%)	196 (23%)	202 (23%)	1580 (26%)	579 (21%)	1001 (32%)	501	260	241
Completed screening	2393 (97%)	994 (96%)	1399 (97%)	396 (99%)	195 (99%)	201 (>99%)	1510 (96%)	545 (94%)	965 (96%)	487 (97%)	254 (98%)	233 (97%)
Ineligible	862 (36%)	286 (29%)	576 (41%)	77 (19%)	27 (14%)	50 (25%)	683 (45%)	209 (38%)	474 (49%)	102 (21%)	50 (20%)	52 (22%)
On ART	66 (8%)	13 (5%)	53 (9%)	5 (6%)	0 (0%)	5 (10%)	58 (8%)	12 (6%)	46 (10%)	3 (3%)	1 (2%)	2 (4%)
CD4 < 100 cells/µL	36 (4%)	21 (7%)	15 (3%)	13 (17%)	7 (26%)	6 (12%)	8 (1%)	2 (1%)	6 (1%)	15 (15%)	12 (24%)	3 (6%)
Pregnant	26 (3%)	0 (0%)	26 (5%)	7 (9%)	0 (0%)	7 (14%)	18 (3%)	0 (0%)	18 (4%)	1 (1%)	0 (0%)	1 (2%)
Creatinine > 133 µmol/L	*14* (2%)	6 (2%)	8 (1%)	3 (4%)	3 (11%)	0 (0%)	9 (1%)	2 (1%)	7 (1%)	2 (2%)	1 (2%)	1 (2%)
WHO Stage 4	3 (<1%)	2 (1%)	1 (<1%)	0 (0%)	0 (0%)	0 (0%)	1 (<1%)	1 (<1%)	0 (0%)	2 (2%)	1 (2%)	1 (2%)
On active TB Tx	6 (1%)	3 (1%)	3 (1%)	0 (0%)	0 (0%)	0 (0%)	6 (1%)	3 (1%)	3 (1%)	0 (0%)	0 (0%)	0 (0%)
Any TB symptoms	76 (9%)	24 (8%)	52 (9%)	9 (12%)	3 (11%)	6 (12%)	67 (10%)	21 (10%)	46 (10%)	0 (0%)	0 (0%)	0 (0%)
Other clinical criteria	1 (<1%)	1 (<1%)	0 (0%)	0 (0%)	0 (0%)	0 (0%)	1 (<1%)	1 (<1%)	0 (0%)	0 (0%)	0 (0%)	0 (0%)
CD4 > 500 cells/µL	36 (4%)	12 (4%)	24 (4%)	36 (47%)	12 (44%)	24 (48%)	0 (0%)	0 (0%)	0 (0%)	0 (0%)	0 (0%)	0 (0%)
Virally suppressed	588 (68%)	199 (70%)	389 (68%)	0 (0%)	0 (0%)	0 (0%)	510 (75%)	165 (79%)	345 (73%)	78 (76%)	34 (68%)	44 (85%)
Refused ART	6 (1%)	4 (1%)	2 (<1%)	3 (4%)	2 (7%)	1 (2%)	2 (<1%)	1 (<1%)	1 (<1%)	1 (1%)	1 (2%)	0 (0%)
Did not consent	4 (<1%)	1 (<1%)	3 (1%)	1 (1%)	0 (0%)	1 (2%)	3 (<1%)	1 (<1%)	2 (<1%)	0 (0%)	0 (0%)	0 (0%)
Enrolled	1531 (64%)	708 (71%)	823 (59%)	319 (81%)	168 (86%)	151 (75%)	827 (55%)	336 (62%)	491 (51%)	385 (79%)	204 (80%)	181 (78%)
Clinic group	514 (34%)	243 (34%)	271 (33%)	99 (31%)	51 (30%)	48 (32%)	269 (33%)	114 (34%)	155 (32%)	146 (38%)	78 (38%)	68 (38%)
Hybrid group	509 (33%)	232 (33%)	277 (34%)	111 (35%)	59 (35%)	52 (34%)	275 (33%)	109 (32%)	166 (34%)	123 (32%)	64 (31%)	59 (33%)
Community group	508 (33%)	233 (33%)	275 (33%)	109 (34%)	58 (35%)	51 (34%)	283 (34%)	113 (34%)	170 (35%)	116 (30%)	62 (30%)	54 (30%)

* Northern KZN SA did extensive pre-screening so the numbers enumerated and tested for HIV are not knowns
KZN SA = KwaZulu-Natal, South Africa

Table S2: Baseline characteristics: intention-to-treat

		Total (n = 1531)		Clinic group (n = 514)		Hybrid group (n = 509)		Community group (n = 508)	
Gender	Male	708	(46%)	168	(53%)	336	(41%)	204	(53%)
	Female	823	(54%)	151	(47%)	491	(59%)	181	(47%)
Age	18 - 29	551	(36%)	121	(38%)	276	(33%)	154	(40%)
	30 - 49	858	(56%)	162	(51%)	493	(60%)	203	(53%)
	> 49	122	(8%)	36	(11%)	58	(7%)	28	(7%)
Education	Primary	441/1497	(29%)	214/304	(70%)	128/817	(16%)	99/376	(26%)
	Secondary	1010/1497	(67%)	75/304	(25%)	662/817	(81%)	273/376	(73%)
	Tertiary	46/1497	(3%)	15/304	(5%)	27/817	(3%)	4/376	(1%)
Employed		594	(39%)	193	(38%)	204	(40%)	197	(39%)
Study household size	1	1462	(95%)	489	(95%)	491	(96%)	482	(95%)
	2	66	(4%)	22	(4%)	18	(4%)	26	(5%)
	3	3	(<1%)	3	(1%)	0	(0%)	0	(0%)
In relationship		982	(64%)	318	(62%)	330	(65%)	334	(66%)
Number of current sex partners	0	140/1521	(9%)	46/508	(9%)	51/506	(10%)	43/507	(8%)
	1	1166/1521	(77%)	387/508	(76%)	384/506	(76%)	395/507	(78%)
	≥ 2	215/1521	(14%)	75/508	(15%)	71/506	(14%)	69/507	(14%)
Condom used at last sex		350/1506	(23%)	125/503	(25%)	107/500	(21%)	118/503	(23%)
Circumcised		153/708	(22%)	56/243	(23%)	47/232	(20%)	50/233	(21%)
Knows of nearby HIV clinic		1498/1530	(98%)	503	(98%)	502/508	(99%)	493	(97%)
WHO stage	Stage 1	1389	(91%)	462	(90%)	465	(91%)	462	(91%)
	Stage 2	118	(8%)	40	(8%)	37	(7%)	41	(8%)
	Stage 3	24	(2%)	12	(2%)	7	(1%)	5	(1%)
CD4 count (cells/μL)	100 - 349	473	(31%)	155	(30%)	151	(30%)	167	(33%)
	350 - 499	370	(24%)	123	(24%)	116	(23%)	131	(26%)
	≥ 500	688	(45%)	236	(46%)	242	(48%)	210	(41%)
Creatinine (μmol/L)	< 106	1452	(95%)	485	(94%)	482	(95%)	485	(95%)
	106 - 133	79	(5%)	29	(6%)	27	(5%)	23	(5%)
DBS viral load (copies/mL)	< 20	216/1420	(15%)	68/472	(14%)	67/471	(14%)	81/477	(17%)
	20 - 999	264/1420	(19%)	89/472	(19%)	96/471	(20%)	79/477	(17%)
	1000 - 9999	476/1420	(34%)	156/472	(33%)	145/471	(31%)	175/477	(37%)
	≥ 10000	464/1420	(33%)	159/472	(34%)	163/471	(35%)	142/477	(30%)

RETENTION

Table S3: Study retention by timepoint

	Enrolled	Month 1		Month 3		Month 6		Month 9		Exit[†]	
	n	n	(%)	n	(%)	n	(%)	n	(%)	n	(%)
**Total**	1531	1270	(83%)	1262	(82%)	1237/1504	(82%)	1120/1377	(81%)	1466	(96%)
Clinic group	514	396	(77%)	405	(79%)	414/503	(82%)	397/465	(85%)	494	(96%)
Hybrid group	509	420	(83%)	405	(80%)	388/502	(77%)	351/458	(77%)	478	(94%)
Community group	508	454	(89%)	452	(89%)	435/499	(87%)	372/454	(82%)	494	(97%)
**SW Uganda**	319	305	(96%)	297	(93%)	263/292	(90%)	125/165	(76%)	287	(90%)
Clinic group	99	98	(99%)	94	(95%)	82/88	(93%)	39/50	(78%)	87	(88%)
Hybrid group	111	100	(90%)	97	(87%)	89/104	(86%)	42/60	(70%)	96	(86%)
Community group	109	107	(98%)	106	(97%)	92/100	(92%)	44/55	(80%)	104	(95%)
**Midlands KZN SA**	827	651	(79%)	648	(78%)	667	(81%)	676	(82%)	810	(98%)
Clinic group	269	203	(75%)	211	(78%)	223	(83%)	235	(87%)	265	(99%)
Hybrid group	275	211	(77%)	198	(72%)	200	(73%)	209	(76%)	266	(97%)
Community group	283	237	(84%)	239	(84%)	244	(86%)	232	(82%)	279	(99%)
**Northern KZN SA**	385	314	(82%)	317	(82%)	307	(80%)	319	(83%)	369	(96%)
Clinic group	146	95	(65%)	100	(68%)	109	(75%)	123	(84%)	142	(97%)
Hybrid group	123	109	(89%)	110	(89%)	99	(80%)	100	(81%)	116	(94%)
Community group	116	110	(95%)	107	(92%)	99	(85%)	96	(83%)	111	(96%)

[†] Includes six participants who did not attend an exit visit but contributed a viral load endpoint via chart abstraction

KZN SA = KwaZulu-Natal, South Africa

ENDPOINTS

Table S4: Sensitivity analyses of relative risks of viral suppression, overall

	Rate of Viral Suppression									Adjusted[*] Relative Risk of Viral Suppression					
	Clinic group			Hybrid group			Community group			Hybrid group vs Clinic group			Community group vs Clinic group		
	N	n	(%)	N	n	(%)	N	n	(%)	RR	(95% CI)	p value	RR	(95% CI)	p value
**mITT (Primary analysis):**<br>*- excluding suppressed at baseline*<br>*- excluding lost-to-follow-up*	446	269/426	(63%)	442	282/413	(68%)	427	306/414	(74%)	1·08	(0·98 - 1·19)	0·12<br>0·0049[†]	1·18	(1·07 - 1·29)	0·00053
**mITT unadjusted:**<br>*- primary model without covariates*	446	269/426	(63%)	442	282/413	(68%)	427	306/414	(74%)	1·08	(0·98 - 1·19)	0·12<br>0·0049[†]	1·17	(1·07 - 1·28)	0·00090
**mITT time adjusted:**<br>*- primary model also adjusted for calendar time (using cubic splines stratified by site, 6 df/year)*	446	269/426	(63%)	442	282/413	(68%)	427	306/414	(74%)	1·10	(1·00 - 1·21)	0·057<br>0·0016[†]	1·19	(1·09 - 1·31)	0·00014
**mITT WHO cut-off:**<br>*- suppression cut-off of < 1000 copies/mL*	446	309/426	(73%)	442	317/413	(77%)	427	348/414	(84%)	1·05	(0·98 - 1·14)	0·18<br>0·0041[†]	1·16	(1·08 - 1·25)	<0·0001
**mITT lost-to-follow-up:**<br>*- excluding suppressed at baseline*<br>*- assuming lost-to-follow-up detectable*	446	269/446	(60%)	442	282/442	(64%)	427	306/427	(72%)	1·06	(0·95 - 1·17)	0·30<br>0·022[†]	1·20	(1·09 - 1·32)	0·00024
**ITT:**<br>*- including suppressed at baseline*<br>*- excluding lost-to-follow-up*	514	322/494	(65%)	509	329/475	(69%)	508	366/491	(75%)	1·06	(0·98 - 1·16)	0·16<br>0·0054[†]	1·15	(1·06 - 1·25)	0·00099
**ITT lost-to-follow-up as detectable:**<br>*- including suppressed at baseline*<br>*- assuming lost-to-follow-up detectable*	514	322/514	(63%)	509	329/509	(65%)	508	366/508	(72%)	1·03	(0·94 - 1·13)	0·50<br>0·039[†]	1·16	(1·06 - 1·26)	0·00087

[*] Adjusted for gender, age less than 30, baseline CD4 count (WHO category), and study site

[†] p value for a one-sided Wald test for non-inferiority (RR > 0·95)

Table S5: Rates and risk differences of viral suppression at exit by group, overall and among subgroups

	Rate of Viral Suppression									Adjusted* Risk Difference of Viral Suppression					
	Clinic group			Hybrid group			Community group			Hybrid group vs Clinic group			Community group vs Clinic group		
	N	n	(%)	N	n	(%)	N	n	(%)	RD	(95% CI)	p value	RD	(95% CI)	p value
**OVERALL**	446	269/426	(63%)	442	282/413	(68%)	427	306/414	(74%)	4·9	(-1·4 - 11·2)	0·13	11·0	(4·8 - 17·1)	0·00048
**Gender**												0·044‡			0·011‡
Male	230	120/221	(54%)	217	134/203	(66%)	219	156/213	(73%)	10·5	(1·2 - 19·8)		18·4	(9·5 - 27·2)	
Female	216	149/205	(73%)	225	148/210	(70%)	208	150/201	(75%)	-1·5	(-10·1 - 7·1)		2·9	(-5·6 - 11·3)	
**Age**												0·90‡			0·58‡
18-29	150	98/144	(68%)	179	114/160	(71%)	158	116/154	(75%)	4·7	(-5·6 - 15·0)		9·1	(-1·2 - 19·4)	
30+	296	171/282	(61%)	263	168/253	(66%)	269	190/260	(73%)	5·0	(-3·0 - 13·1)		12·3	(4·5 - 20·0)	
**Site**												0·22‡			0·16‡
SW Uganda	96	64/84	(76%)	108	66/91	(73%)	101	74/94	(79%)	-2·3	(-15·4 - 10·8)		4·1	(-8·3 - 16·6)	
Midlands KZN SA	217	125/213	(59%)	221	137/216	(63%)	222	151/220	(69%)	4·4	(-4·7 - 13·5)		9·8	(0·9 - 18·8)	
Northern KZN SA	133	80/129	(62%)	113	79/106	(75%)	104	81/100	(81%)	12·0	(0·5 - 23·6)		19·2	(7·9 - 30·6)	
**SOUTH AFRICA**	350	205/342	(60%)	334	216/322	(67%)	326	232/320	(72%)	7·0	(-0·2 - 14·2)	0·056	12·9	(5·9 - 20·0)	0·00032
**Gender**												0·048‡			0·025‡
Male	181	91/178	(51%)	161	100/153	(65%)	163	113/158	(72%)	13·1	(2·5 - 23·7)		20·1	(10·0 - 30·3)	
Female	169	114/164	(70%)	173	116/169	(69%)	163	119/162	(73%)	-0·3	(-10·2 - 9·5)		4·7	(-5·0 - 14·5)	

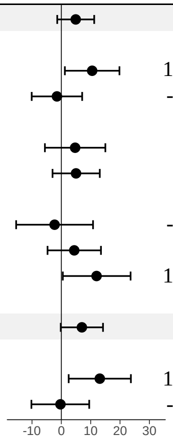

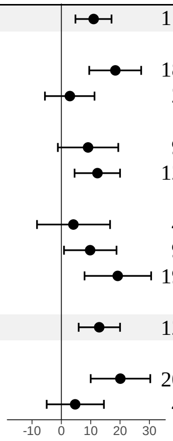

* Adjusted for gender, age less than 30, baseline CD4 count (WHO category), and study site
‡ p value for a Wald test for significant interaction
KZN SA = KwaZulu-Natal, South Africa

Table S6: Rates and relatives risks of viral suppression at exit by group and gender

	Rate of Viral Suppression									Adjusted* Relative Risk of Viral Suppression			
	Clinic group			Hybrid group			Community group			Hybrid group vs Clinic group		Community group vs Clinic group	
	N	n	(%)	N	n	(%)	N	n	(%)	RR (95% CI)	p value	RR (95% CI)	p value
**SW Uganda**	96	64/84	(76%)	108	66/91	(73%)	101	74/94	(79%)	0·97 (0·81 - 1·16)	0·73	1·06 (0·90 - 1·24)	0·51
											0·41†		0·10†
**Gender**											0·55‡		0·17‡
Male	49	29/43	(67%)	56	34/50	(68%)	56	43/55	(78%)	1·00 (0·75 - 1·34)		1·16 (0·90 - 1·50)	
Female	47	35/41	(85%)	52	32/41	(78%)	45	31/39	(79%)	0·97 (0·79 - 1·18)		0·95 (0·78 - 1·15)	
**Midlands KZN SA**	217	125/213	(59%)	221	137/216	(63%)	222	151/220	(69%)	1·08 (0·93 - 1·25)	0·34	1·17 (1·01 - 1·35)	0·034
											0·053†		
**Gender**											0·024‡		0·074‡
Male	108	53/106	(50%)	101	65/99	(66%)	106	71/105	(68%)	1·29 (1·02 - 1·64)		1·34 (1·06 - 1·69)	
Female	109	72/107	(67%)	120	72/117	(62%)	116	80/115	(70%)	0·92 (0·76 - 1·12)		1·04 (0·87 - 1·24)	
**Northern KZN SA**	133	80/129	(62%)	113	79/106	(75%)	104	81/100	(81%)	1·20 (1·01 - 1·42)	0·041	1·31 (1·11 - 1·55)	0·0012
**Gender**											0·74‡		0·11‡
Male	73	38/72	(53%)	60	35/54	(65%)	57	42/53	(79%)	1·17 (0·87 - 1·58)		1·52 (1·17 - 1·97)	
Female	60	42/57	(74%)	53	44/52	(85%)	47	39/47	(83%)	1·17 (0·96 - 1·42)		1·14 (0·93 - 1·39)	

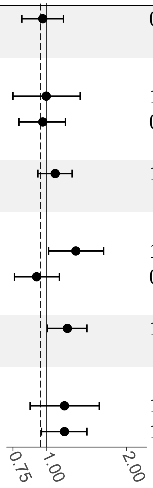

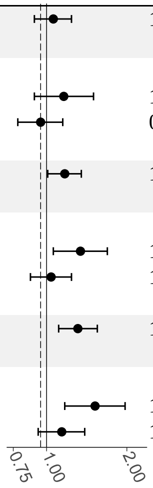

* Adjusted for gender, age less than 30, baseline CD4 count (WHO category), and study site

† p value for a one-sided Wald test for non-inferiority (RR > 0·95). The relative non-inferiority margin is represented by a dashed line in the forest plot.

‡ p value for a Wald test for significant interaction

Table S7: Severe adverse events and social harms – participant-level summary by group

	Total (n = 1531)		Clinic group (n = 514)		Hybrid group (n = 509)		Community group (n = 508)	
Serious adverse events	20	(1·31%)	8	(1·56%)	5	(0·98%)	7	(1·38%)
Grade 5	9	(0·59%)	4	(0·78%)	2	(0·39%)	3	(0·59%)
Grade 4	1	(0·07%)	1	(0·19%)	0	(0·00%)	0	(0·00%)
Grade 3	9	(0·59%)	3	(0·58%)	2	(0·39%)	4	(0·79%)
Grade 2	1	(0·07%)	0	(0·00%)	1	(0·20%)	0	(0·00%)
Severe adverse events	13	(0·85%)	2	(0·39%)	4	(0·79%)	7	(1·38%)
Grade 3	13	(0·85%)	2	(0·39%)	4	(0·79%)	7	(1·38%)
Social harms	2	(0·13%)	0	(0·00%)	0	(0·00%)	2	(0·39%)

COST ANALYSIS DETAILS

Cost Estimation

We estimated the annual per-client cost of community-based ART care from the provider's perspective. We report separate cost estimates for the first year of ART (including initiation and follow-up visits at 1, 3, 6, and 9 months) and additional years of ART (consisting of four three-month follow-up visits). Costs include all activities supporting ART initiation and follow-up; we excluded outreach HIV testing costs and research-specific procedures.

Community ART delivery was implemented differently at each site. At ICOBI in southwest Uganda, field workers generally travelled to visit sites by motorcycle, which allowed easier access to rural sites, or by pickup truck with a dedicated driver. In South Africa, field workers were organized into teams of two or three individuals traveling by pickup truck or panel van. Each team included a nurse trained in ART initiation and management, an assistant, and occasionally a separate driver. We used field observation, interviews, time-motion studies, and standardized logs to measure resources consumed by each field team while implementing community ART delivery.

We categorized costs as either fixed (constant over the course of one year) or variable (directly related to the number of client encounters). We obtained drug prices from international reference price lists for Uganda and the South Africa Department of Health procurement catalogue for South Africa.[1–3] Laboratory monitoring costs included point-of-care CD4 testing at initiation, annual creatinine testing, and semi-annual viral load testing. We estimated fuel costs using mileage from vehicle trip logs and local fuel prices. Fixed costs were categorized as start-up, equipment, building, and personnel costs. Start-up costs included personnel, venue, and material costs incurred during hiring, training, and community mobilization. Equipment included vehicles and maintenance, PIMA CD4 analysers, furniture, uniforms, and computers. Vehicles for ART delivery included motorcycles (Uganda), pickup trucks (all sites), and modified panel vans (South Africa). We assumed a useful life of five years for motorcycles and ten years for trucks and vans. We annualized start-up, vehicle, and equipment costs over the expected useful life using a discount rate of 3%.[4] We estimated building and utilities costs using a rental rate derived from nearby commercial properties. Personnel salaries were derived from study budgets and included both clinical and administrative staff. We allocated administrative staff salaries based on the fraction of full-time equivalents spent supporting ART delivery (ascertained through interviews). We converted all costs to US dollars using the average local exchange rate over the relevant year and adjusted all to 2018 USD using GDP deflators.[5]

Scenarios

The total number of clients in the DO ART Study was determined by statistical considerations for the primary endpoint rather than program capacity, and research salaries may not reflect personnel costs in programmatic settings. As such, we estimated cost per client by varying these assumptions in three scenarios. In the first scenario (steady-state, as observed), we used costs as observed in the study but assumed that the maximum client volume achieved during the DO ART Study was sustained for 12 months. In the second scenario (steady-state, programmatic), we used the steady-state client volume but substituted salaries paid under the research study with representative salaries derived from Ministry of Health salary scales. This scenario may more closely reflect personnel costs under local implementation. In the third scenario (efficient at-scale), we used time-motion studies to estimate the average duration of client encounters conducted by field teams at each site during peak client volume during the study. We then calculated the maximum number of clients that could be seen in an eight-hour workday after removing travel, administrative, and break time. We estimated the total number of clients that could be seen in 12 months assuming that the maximum number of encounters was achieved each day (Table S8 and Table S9). This scenario assumes no intervening travel time between client encounters and is intended to represent a mobile unit servicing one community location per day and operating at full capacity.

Table S8: Estimated client volume by site for efficient at-scale scenario

	SW Uganda	Midlands KZN SA	Northern KZN SA
Average time per visit (hours)	0·84	0·5	0·6
Number of visits per day per team	6·8	13·1	10·5
Number of clients per year per team	328	629	505
Number of teams*	5	3	3
Number of clients per year	1638	1887	1514

*As observed during peak client volume
KZN SA = KwaZulu-Natal, South Africa

Table S9: Estimated number of clients seen per year per under steady-state and efficient at-scale scenarios

	SW Uganda	Midlands KZN SA	Northern KZN SA
Steady State	218	714	720
Efficient	1638	1887	1541

Table S10: Unit costs of key inputs (2018 USD)

	SW Uganda	Midlands KZN SA	Northern KZN SA
ART (per year)	102[1]	114[2]	114[2]
Trimethoprim/sulfamethoxazole (per year)	8·0[3]	4·19[2]	4·19[2]
Viral load (per test)	20·18[*]	26·02[*]	26·02[*]
CD4 cartridge (per test)	8·69[*]	6·81[*]	6·84[*]
Creatinine (per test)	1·52[*]	7·96[*]	7·96[*]
HIV (per test)	0·87[*]	0·55[*]	0·74[*]
Fuel (average cost per visit)	3·35[*]	0·87[*]	0·73[*]
Nurse (salary per month – research)	2500[*]	1446[*]	2866[*]
Nurse (salary per month – MOH)	264[4]	2064[5]	2027[5]

[*]Obtained from study budgets

Annual cost per client

Using the estimated costs and volumes described above, we calculated the annual cost per client at each site for each scenario (Table S10-S12). We divided these costs by the proportion suppressed at 12 months observed in the study to estimate the cost per client virally suppressed (Table S13). The cost per client virally suppressed for subsequent years of ART assume that the proportion suppressed is constant.

Table S11: Annual cost per client in steady-state (as observed) scenario (2018 USD)

		First Year						Subsequent Years					
		SW Uganda		Midlands KZN SA		Northern KZN SA		SW Uganda		Midlands KZN SA		Northern KZN SA	
		Cost	(%)	Cost	(%)	Cost	(%)	Cost	(%)	Cost	(%)	Cost	(%)
Variable	Drugs[*]	110	(7%)	118	(19%)	118	(21%)	110	(9%)	118	(23%)	118	(26%)
	Labs	36	(2%)	60	(9%)	60	(10%)	22	(2%)	34	(7%)	34	(7%)
	Fuel	17	(1%)	4	(1%)	4	(1%)	13	(1%)	3	(1%)	2	(<1%)
	Other	2	(<1%)	1	(<1%)	1	(<1%)	2	(<1%)	1	(<1%)	1	(<1%)
Variable Total		*165*	*(11%)*	*183*	*(29%)*	*183*	*(32%)*	*148*	*(12%)*	*156*	*(30%)*	*156*	*(34%)*
Fixed	Personnel	1142	(73%)	377	(59%)	319	(56%)	913	(74%)	302	(58%)	256	(55%)
	Vehicles	83	(5%)	24	(4%)	25	(4%)	66	(5%)	19	(4%)	20	(4%)
	Equipment	38	(2%)	5	(1%)	8	(1%)	5	(<1%)	1	(<1%)	1	(<1%)
	Building	76	(5%)	21	(3%)	15	(3%)	61	(5%)	17	(3%)	12	(3%)
	Start-up	62	(4%)	27	(4%)	24	(4%)	50	(4%)	22	(4%)	19	(4%)
Fixed Total		*1400*	*(89%)*	*454*	*(71%)*	*391*	*(68%)*	*1095*	*(88%)*	*361*	*(70%)*	*308*	*(66%)*
**Total**		**1565**	**(100%)**	**637**	**(100%)**	**574**	**(100%)**	**1243**	**(100%)**	**516**	**(100%)**	**464**	**(100%)**

[*]Drug costs include ART ($114/year in South Africa, $102 in Uganda) and co-trimoxazole ($4/year in South Africa, $8/year in Uganda)

Table S12: Annual cost per client in steady-state (programmatic) scenario (2018 USD)

		First Year						Subsequent Years					
		SW Uganda		Midlands KZN SA		Northern KZN SA		SW Uganda		Midlands KZN SA		Northern KZN SA	
		Cost	(%)	Cost	(%)	Cost	(%)	Cost	(%)	Cost	(%)	Cost	(%)
Variable	Drugs[*]	110	(20%)	118	(23%)	118	(27%)	110	(25%)	118	(28%)	118	(33%)
	Labs	36	(6%)	60	(11%)	60	(13%)	22	(5%)	34	(8%)	34	(9%)
	Fuel	17	(3%)	4	(1%)	4	(1%)	13	(3%)	3	(1%)	3	(1%)
	Other	2	(<1%)	1	(<1%)	1	(<1%)	2	(<1%)	1	(<1%)	1	(<1%)
Variable Total		*165*	*(30%)*	*183*	*(35%)*	*183*	*(41%)*	*148*	*(34%)*	*157*	*(37%)*	*157*	*(43%)*
Fixed	Personnel	176	(32%)	262	(50%)	190	(43%)	141	(32%)	210	(49%)	152	(42%)
	Vehicles	83	(15%)	24	(5%)	25	(6%)	66	(15%)	19	(5%)	20	(5%)
	Equipment	38	(7%)	5	(1%)	8	(2%)	5	(1%)	1	(<1%)	1	(<1%)
	Building	76	(14%)	21	(4%)	15	(3%)	61	(14%)	17	(4%)	12	(3%)
	Start-up	19	(3%)	29	(6%)	26	(6%)	15	(3%)	23	(5%)	20	(6%)
Fixed Total		*391*	*(70%)*	*341*	*(65%)*	*263*	*(59%)*	*288*	*(66%)*	*270*	*(63%)*	*205*	*(57%)*
**Total**		**555**	**(100%)**	**523**	**(100%)**	**446**	**(100%)**	**435**	**(100%)**	**427**	**(100%)**	**362**	**(100%)**

[*]Drug costs include ART ($114/year in South Africa, $102 in Uganda) and co-trimoxazole ($4/year in South Africa, $8/year in Uganda)

Table S13: Annual cost per client in efficient at-scale scenario (2018 USD)

		First Year						Subsequent Years					
		SW Uganda		Midlands KZN SA		Northern KZN SA		SW Uganda		Midlands KZN SA		Northern KZN SA	
		**Cost**	**(%)**	**Cost**	**(%)**	**Cost**	**(%)**	**Cost**	**(%)**	**Cost**	**(%)**	**Cost**	**(%)**
Variable	Drugs*	110	(51%)	118	(38%)	118	(38%)	110	(59%)	118	(48%)	118	(49%)
	Labs	36	(16%)	60	(19%)	60	(19%)	22	(12%)	34	(14%)	34	(14%)
	Fuel	17	(8%)	4	(1%)	4	(1%)	13	(7%)	3	(1%)	3	(1%)
	Other	2	(<1%)	1	(<1%)	1	(<1%)	2	(1%)	1	(<1%)	1	(1%)
Variable Total		*165*	*(76%)*	*183*	*(59%)*	*183*	*(59%)*	*148*	*(79%)*	*157*	*(64%)*	*157*	*(64%)*
Fixed	Personnel	23	(11%)	99	(32%)	90	(29%)	19	(10%)	69	(28%)	64	(26%)
	Vehicles	11	(5%)	9	(3%)	12	(4%)	9	(5%)	6	(3%)	8	(3%)
	Equipment	5	(2%)	2	(1%)	4	(1%)	1	(<1%)	<1	(<1%)	<1	(<1%)
	Building	10	(5%)	8	(3%)	7	(2%)	8	(4%)	6	(2%)	5	(2%)
	Start-up	3	(1%)	11	(3%)	12	(4%)	2	(1%)	8	(3%)	9	(4%)
Fixed Total		*52*	*(24%)*	*129*	*(41%)*	*125*	*(41%)*	*40*	*(21%)*	*89*	*(36%)*	*87*	*(36%)*
**Total**		**217**	**(100%)**	**312**	**(100%)**	**308**	**(100%)**	**187**	**(100%)**	**246**	**(100%)**	**244**	**(100%)**

*Drug costs include ART ($114/year in South Africa, $102 in Uganda) and co-trimoxazole ($4/year in South Africa, $8/year in Uganda)

Table S14: Estimated annual costs per client and per client virally suppressed under community-based ART delivery (2018 USD)

		First Year			Subsequent Years		
	**Scenario**	**SW Uganda**	**Midlands KZN SA**	**Northern KZN SA**	**SW Uganda**	**Midlands KZN SA**	**Northern KZN SA**
**Annual cost per client**	Steady-state (as observed)	1565	637	574	1243	516	464
	Steady-state (programmatic)	555	523	446	435	427	362
	Efficient at-scale	217	312	308	187	246	244
**Annual cost per client virally suppressed**	Steady-state (as observed)	1981	923	709	1702	820	618
	Steady-state (programmatic)	703	759	551	596	678	483
	Efficient at-scale	274	452	380	257	390	325

We did not measure costs incurred among clients receiving clinic-based care. Instead, we estimated the cost per client virally suppressed by combining viral suppression rates in the clinic-based arm with recently published cost estimates for annual adult ART costs in South Africa.[6] We compared these to the estimates from the efficient scenario using first-year ART costs (Table S14).

Table S15: Comparison of estimated community-based (efficient at-scale scenario) vs. facility-based annual cost per client virally suppressed in South Africa and Uganda

	SW Uganda		Midlands KZN SA		Northern KZN SA	
	**Facility**	**Community**	**Facility**	**Community**	**Facility**	**Community**
Annual cost per client	$163**[6]	$217	$249[7]	$312*	$249[7]	$308*
% virally suppressed	76%	79%	59%	69%	62%	81%
Annual cost per client virally suppressed	$214	$275	$422	$452	$402	$380

*Cost under efficient at-scale scenario

*Cost under maximally streamlined scenario (scenario C)[6]

Limitations

The analyses described above have several limitations. First, the cost estimates beyond the first year are projected based on what was observed during the first year and could change. Costs could decrease if visit durations shorten over time, which may occur as clients become more familiar with ART; alternatively, factors such as resistance and dropout could increase costs. Second, the cost projections under the efficient at-scale scenarios require sufficient volume of clients. Ministries of Health would need to negotiate with clinical staff to work in a new environment to provide these services. These factors were less important with lower client volumes and research staff in the DO ART Study. The scenario analysis results represent potential feasibility as well as general guidance on the inputs and volumes necessary for community-based delivery costs to be similar to facility-based costs. Implementation studies of community-based ART delivery outside of the setting of randomized trials are needed to better define the feasibility, cost, and scalability of delivery models.

EVOLVING STANDARD OF CARE

The Kabwohe Clinical Research Centre ART clinic is a private not-for-profit health facility located in southwest Uganda and is supported by the United States Agency for International Development and the Elizabeth Glaser Pediatric AIDS Foundation. The partner clinics in Midlands KZN and Northern KZN, South Africa are provincial health clinics operated by the KwaZulu-Natal Department of Health.

Table S16. Summary of changes in standard of care by site

	SW Uganda		Midlands KZN SA		Northern KZN SA	
**Practice**	**Date started**	**Description**	**Date started**	**Description**	**Date started**	**Description**
Treat at any CD4	Dec-2016	National guidelines changed to treat at any CD4 count.	Pre-DO ART	National guidelines changed to treat at any CD4 count.	Pre-DO ART	National guidelines changed to treat at any CD4 count.
Same day ART initiation	Dec-2016	With the removal of the CD4 criteria for ART start, the clinic stopped requiring multiple pre-ART visits. The client is assessed for readiness to start ART (same-day CD4 and OI assessment). Initial dispensation of ART is 15 days.	Sep-2017	Department of Health guidelines change, all clinics are obliged to follow "same-day test and treat" policy. Baseline labs are collected the same day a client tests positive.	Sep-2017	Department of Health guidelines change, all clinics are obliged to follow "same-day test and treat" policy. Baseline labs are collected the same day a client tests positive.
Alternative options for ART dispensing	Jun-2017	Once clients have one suppressed viral load (at 6 months post ART start), they are given the option to either be put into a group with 3 others all from the same location, rotating picking up ART, or dispensed at the pharmacy with 6 months refill (half dispensed immediately and half dispensed three months later without requiring a nurse visit.	Jul-2017	Medically stable clients are put onto the Medipost program. Clients in this program go straight to the Medipost section of the clinic and pick up their medication, which only takes 5 to 15 minutes.	2017	Once clients have a suppressed viral load (at 12 months post ART start), they can transition into CCMDD which allows for monthly ART pick-up at the in-clinic pharmacy, without having to see a care provider. Clients see a provider every 6 months.

Table S17. Standard of care description at Kabwohe Clinical Research Centre (SW Uganda)

Practice	Date Started	Description	Date Modified	Reason for Change
Pre-ART visits	Pre-DO ART	Multiple visits were required prior to ART start, to assess eligibility and willingness.	Dec-2016	CD4 guidelines change and roll-out of same day start
Intensive adherence counselling	Pre-DO ART	Individuals with unsuppressed viral loads are contacted to return to the clinic for intensive adherence counselling.	Continuing	N/A
Treat at any CD4	Dec-2016	National guidelines changed to treat at any CD4 count.	Continuing	N/A
Same day ART initiation	Dec-2016	With the removal of the CD4 criteria for ART start, the clinic stopped requiring multiple pre-ART visits. The patient is assessed for readiness to start ART (same-day CD4 and OI assessment). Initial dispensation of ART is 15 days.	Continuing	N/A
ART clubs (clusters)	Jun-2017	Once clients have one suppressed viral load (at six months post ART start), they are given the option to either be put into a group with 3 others all from the same location, rotating picking up ART, or dispensed at the pharmacy with 6 months refill (half dispensed immediately and half dispensed three months later without requiring a nurse visit).	Continuing	N/A
Family tracking tools	Aug-2017	Once an HIV-positive client initiates care at the clinic, a family tracking tool is also filled to capture all family members. This helps the clinic encourage the patient to bring all other family members for HIV testing services and contributes to early identification of cases.	Continuing	N/A
Discordant couples meetings	Sep-2017	Discordant couples are clearly identified and given same-day appointments where they meet as a group and are counselled on prevention and adherence, share challenges and successes on disclosure, in addition to other positive living practices.	Continuing	N/A
Viral load testing visit reminders	Oct-2017	Clients who are due for viral load testing are reminded the day before their appointment to ensure no missed visits.	Continuing	N/A

Table S18. Standard of care description at Caluza, Mpumuza, and Pata clinics (Midlands KZN SA)

Practice	Date Started	Description	Date Modified	Reason for Change
Treat at any CD4	Pre-DO ART	National guidelines changed to treat at any CD4 count.	Continuing	N/A
ART clubs	Pre-DO ART	Community-based ART clubs. Each club is run slightly differently depending upon clinic.	Continuing	N/A
Doctor review	Pre-DO ART	ART patients are only reviewed by the doctor if there are complications, otherwise are seen by a nurse.	Continuing	N/A
Fast track at clinic	Jul-2017	Medically stable clients are all put onto the Medipost program. Clients in this program go straight to the Medipost section of the clinic and pick up their medication, which only takes 5 to 15 minutes.	Continuing	N/A
Same day ART initiation	Sep-2017	Department of Health guidelines change, all clinics are obliged to follow "same-day test and treat" policy. Baseline labs are collected the same day a client tests positive.	Continuing	N/A

Table S19. Standard of care description at Ashdown clinic (Midlands KZN SA)

Practice	Date Started	Description	Date Modified	Reason for Change
Treat at any CD4	Pre-DO ART	National guidelines changed to treat at any CD4 count.	Continuing	N/A
ART clubs	Jan-2015	There is one community-based ART club that is run by a facilitator and caregivers. Clients can pick up their refills during fast track hours (8-10 am) and skip lines.	Continuing	N/A
Doctor review	Pre-DO ART	ART patients are only reviewed by the doctor if there are complications.	Continuing	N/A
Fast track at clinic	Jul-2017	Medically stable clients are put onto the Medipost program. Clients in this program go straight to the Medipost section of the clinic and pick up their medication, which only takes 5 to 15 minutes.	Oct-17	4-month trial, demand was not high enough
Same day ART Start	Dec-2017	Department of Health guideline change, all clinics are obliged to follow "same-day test and treat" policy. Baseline labs are collected the same day a client tests positive.	Continuing	N/A

Table S20. Standard of care description at Nkundusi, Madwaleni, Ntondweni, and Zwenelisha clinics (Northern KZN SA)

Practice	Date Started	Description	Date Modified	Reason for Change
Treat at any CD4	Pre-DO ART	National guidelines changed to treat at any CD4 count.	Continuing	N/A
Fast Track at Clinic	Pre-DO ART	Clients who are current students, geriatric, or very ill can go to the front of the line and be served first	Continuing	N/A
Same day ART Start	Sep-2017	Department of Health guideline change, all clinics are obliged to follow "same-day test and treat" policy. Baseline labs are collected the same day a client tests positive.	Continuing	N/A
CCMDD	2017	All clients virally suppressed at 12 months move into CCMDD. CCMDD pickup only available in clinic.	Continuing	N/A

Table S21. Standard of care description at Sipho Zungu clinic (Northern KZN SA)

Practice	Date Started	Description	Date Modified	Reason for Change
Treat at any CD4	Pre-DO ART	National guidelines changed to treat at any CD4 count.	Continuing	N/A
Same day ART Start	Sep-2017	As per new Department of Health guidelines, all clinics are obliged to follow "same-day test and treat" policy. Baseline labs are collected the same day a client tests positive.	Continuing	N/A
CCMDD	2017	All clients virally suppressed at 12 months move into CCMDD, CCMDD pickup only available in clinic.	Continuing	N/A

REFERENCES

1. Clinton Health Access Initiative. The State of the Antiretroviral Drug Market in Low- and Middle-Income Countries, 2015-2020., 2016.
2. South Africa Department of Health. Medicine Procurement List. 2017.
3. Management Sciences for Health. International Medical Products Price Guide, 2015.
4. Uganda Ministry of Public Health. Salary Structure for Medical Workers.
5. Department of Public Service and Administration RoSA. Salary scales, with translation keys, for employees on salary levels 1 to 12 and those employees covered by Occupation Specific Dispensations (OSDs). 2016.
6. Shade SB, Osmand T, Luo A, et al. Costs of streamlined HIV care delivery in rural Ugandan and Kenyan clinics in the SEARCH Studys. *Aids* 2018; **32**(15): 2179-88.
7. Meyer-Rath G, van Rensburg C, Chiu C, Leuner R, Jamieson L, Cohen S. The per-patient costs of HIV services in South Africa: Systematic review and application in the South African HIV Investment Case. *PloS one* 2019; **14**(2): e0210497.
